# Circular RNA circLIFR suppresses papillary thyroid cancer progression by modulating the miR-429/TIMP2 axis

**DOI:** 10.1007/s00432-024-05839-7

**Published:** 2024-06-25

**Authors:** Fengyuan Zhang, Jiazheng Li, Jingjing Xu, Xugan Jiang, Shengxia Chen, Qais Ahmad Nasser

**Affiliations:** Department of Laboratory Medicine, School of Medicine, Jiangsu University, Zhenjiang, 212013 China

**Keywords:** Circular RNA, CircLIFR, Papillary thyroid cancer, miR-429, TIMP2

## Abstract

**Purpose:**

Circular RNAs (circRNAs) are increasingly recognized for their important roles in various cancers, including papillary thyroid cancer (PTC). The specific mechanisms by which the circLIF receptor subunit alpha (circLIFR, hsa_circ_0072309) influences PTC progression remain largely unknown.

**Methods:**

In our study, CircLIFR, miR-429, and TIMP2 levels were assessed using reverse transcription-quantitative PCR. The roles of circLIFR and miR-429 in PTC cells were determined using Cell Counting Kit-8, colony formation, wound healing, and Transwell assays. Western blotting was utilized to examine the levels of TIMP2. The direct interaction between circLIFR, TIMP2, and miR-429 was confirmed using dual-luciferase reporter, RNA immunoprecipitation, and fluorescence in situ hybridization assays.

**Results:**

In PTC tissues and cells, a decrease in circLIFR and TIMP2 levels, accompanied by an increase in miR-429 levels, was observed. Overexpression of circLIFR or downregulation of miR-429 effectively suppressed the proliferation and migration of PTC cells. Conversely, the knockdown of circLIFR or overexpression of miR-429 had the opposite effect. Furthermore, circLIFR overexpression suppressed tumor growth in vivo. Mechanistically, circLIFR modulated TIMP2 expression by serving as a sponge for miR-429. Rescue experiments indicated that the antitumor effect of circLIFR could be reversed by miR-429.

**Conclusion:**

This study confirmed circLIFR as a novel tumor suppressor delayed PTC progression through the miR-429/TIMP2 axis. These findings suggested that circLIFR held promise as a potential therapeutic target for PTC.

**Supplementary Information:**

The online version contains supplementary material available at 10.1007/s00432-024-05839-7.

## Introduction

Thyroid cancer is the predominant tumor of the endocrine system (Siegel et al. 2023). In recent decades, the rise in thyroid cancer incidence has markedly outpaced that of other cancers, with 586,202 new cases reported globally in 2020 (Sung et al. 2021). Notably, the incidence rate among women is approximately three times that among men (Sung et al. 2021). Papillary thyroid cancer (PTC) stands out as the most prevalent subtype of differentiated thyroid cancer, constituting ~ 85% of all cases (Schlumberger and Leboulleux 2021). The majority of patients with PTC have favorable outcomes following surgical resection and radioactive iodine therapy (Kreissl et al. 2019; Agosto Salgado et al. 2023; Cao et al. 2021). Nonetheless, a minority of patients exhibit recurrent or metastatic disease, resulting in reduced survival rates (Kreissl et al. 2019; Agosto Salgado et al. 2023; Cao et al. 2021). This highlights the need to identify novel biomarkers and elucidate their molecular mechanisms to improve patient survival.

Circular RNAs (circRNAs) are a distinct class of non-coding RNAs, characterized by their covalent closed circular structure formed via back splicing (Li et al. 2020). They are ubiquitously present in eukaryotes (Rybak-Wolf et al. 2015). Contrasting with mRNAs, circRNAs lack both a 5’ cap and a 3’ poly(A) tail structure (Liu et al. 2019). Furthermore, their resilience against nucleic acid exonuclease renders them less prone to degradation (Suzuki et al. 2006). Due to these unique attributes, circRNAs are emerging as promising biomarker candidates. A growing body of research has demonstrated the pivotal role of circRNAs in the initiation and progression of diverse cancer types and their influence on cellular processes, including proliferation, apoptosis, migration, and invasion, has been rigorously documented (Liu et al. 2019). This beckons further exploration of the involvement of circRNAs in PTC and their potential molecular pathways.

The present study identified a circRNA, hsa_circ_0072309 [referred to as circLIF receptor subunit alpha (LIFR)], originating from the LIFR gene locus. CircLIFR was markedly downregulated in PTC cells and tissues. The present study indicated that circLIFR overexpression inhibited PTC cell migration and proliferation, whereas circLIFR knockdown had the opposite effect. Mechanistically, circLIFR acted as a sponge for microRNA (miRNA/miR)-429 to up-regulate TIMP metallopeptidase inhibitor 2 (TIMP2) expression, thereby inhibiting the proliferation and migration of PTC cells.

## Materials and methods

### Clinical tissue samples

Between July 2021 and December 2022, 53 pairs of PTC and adjacent normal tissues were collected from patients with PTC undergoing surgery at the Affiliated Hospital of Jiangsu University (Zhenjiang, China). Inclusion criteria involved patients with a pathological confirmation of PTC. Conversely, patients with a confirmed diagnosis of PTC who had received alternative treatments before surgery were excluded. There were 12 males and 41 females. The mean age of these patients was 47.81 ± 12.5 years (*n* = 53). All participants were informed about the study and signed informed consent forms. Specimens were confirmed by histopathological biopsy and verified by pathologists. The Ethics Committee of Jiangsu University (Zhenjiang, China) approved the present study, adhering to government policies and The Declaration of Helsinki (version 2013).

### Bioinformatics analysis

GSE171011 and GSE168449 from the Gene Expression Omnibus (GEO) database were analyzed using the DESeq2 package in R software (version 4.1.3; https://www.r-project.org/). Encyclopedia of RNA Interactomes (ENCORI) (https://rnasysu.com/encori/index.php) was used to predict the miRNAs that might bind to circLIFR. TargetScan (https://www.targetscan.org/vert_80/), miRDB (http://www.mirdb.org), TarBase (https://dianalab.e-ce.uth.gr/tarbasev8), and miRTarBase (https://mirtarbase.cuhk.edu.cn) were used to predict the downstream target genes of miR-429.

### Cell culture and transfection

Human PTC cell line TPC-1, thyroid cancer cell line B-CPAP, and normal thyroid epithelial cell line Nthy-ori3-1, provided by the cell bank of type culture collection of the Chinese Academy of Sciences, were cultured in RPMI 1640 medium containing 10% fetal bovine serum (both from Shanghai XP Biomed Ltd.) and 1% penicillin-streptomycin (Biosharp Life Sciences). PTC cell line GLAG-66 cells, supplied by Shanghai EK-Bioscience Biotechnology Co., Ltd, were cultured in DMEM high glucose medium containing 10% fetal bovine serum (Shanghai XP Biomed Ltd.) and 1% penicillin-streptomycin (Biosharp Life Sciences). All cells were maintained at 37 ˚C in an atmosphere with 5% CO_2_. Small interfering RNA (siRNA/si)-circLIFR and miR-429 mimic/inhibitor were designed and synthesized by Suzhou GenePharma Co., Ltd. (Table S1). Cells were transfected with si-circLIFR (50 nM) or miR-429 mimic/inhibitor (50 nM) at 37 ˚C for 8 h using Lipofectamine 3000 (Invitrogen; Thermo Fisher Scientific, Inc.). The time interval between transfection and subsequent experimentation was 48 h.

### Construction of stable cell lines

The overexpression of circLIFR was achieved by integrating its sequence into the pLC5-ciR lentiviral vector (Geneseed Biotech Co., Ltd). 1 × 10^6^ TPC-1 and GLAG-66 cells were infected with the resultant lentivirus (MOI = 50) for 8 h. 48 h after infection, cells were cultured in a complete medium supplemented with 2 µg/ml puromycin (Biosharp Life Sciences) for 2 weeks to establish stable cell lines. Subsequently, the cells were maintained in a complete medium supplemented with 0.5 µg/ml puromycin and subsequent experiments were performed 48 h post-transduction.

### RNA extraction, RNase R treatment and reverse transcription-quantitative PCR (RT-qPCR)

Total RNA was extracted from cells or tissues using the RNA-easy isolation reagent (Vazyme Biotech Co., Ltd.). Nuclear and cytoplasmic RNA separation was performed according to a previous protocol (Chen et al. 2019) using a specific kit (Beyotime Institute of Biotechnology). For RNase R treatment, RNA was incubated with RNase R (LGC Biosearch Technologies) at 37 ℃ for 20 min. RT-PCR and qPCR were conducted using the HiScript III RT SuperMix and AceQ Universal SYBR qPCR Master Mix from Vazyme Biotech Co., Ltd., respectively. PCR reaction protocol was as follows: for RT-PCR, 37 ℃ for 15 min and 85 ℃ for 5 s; for qPCR, pre-denaturation at 95 ℃ for 5 min, 40 cycles of 95 ℃ for 10 s, 60 ℃ for 30 s. GAPDH and U6 served as internal references for circRNA/mRNA and miRNA, respectively. The 2^−ΔΔCt^ method was used to analyze relative RNA expression. All primers (Sangon Biotech Co., Ltd.) are shown in Tables S2 and S3.

### Fluorescence in situ hybridization (FISH)

FISH was performed using a FISH kit from Suzhou GenePharma Co., Ltd. Probes, designed and synthesized by Suzhou GenePharma Co., Ltd., targeted circLIFR (Cy-3 labeled) and miR-429 (5’-FAM labeled). Probe sequences were as follows: circLIFR: 5’-TCT GTG CAA TGC AGT CAG TCT AAT TTT ACG AGC TCC ATA C-3’; miR-429: 5’-ACG GTT TTA CCA GAC AGT ATT A-3’. 1 × 104 TPC-1 and GLAG-66 cells were cultured overnight after seeding onto coverslips, fixed with 4% paraformaldehyde for 15 min at room temperature, and treated with 0.1% Triton X-100 at room temperature for 15 min. After washing twice with PBS, 100 µl 2×Saline Sodium Citrate buffer (SSC) was added to each well and incubated at 37 ℃ for 30 min. The probe (2 µM) was mixed with hybridization buffer and denatured at 75 ℃ for 5 min. The cells were incubated with 200 µl of the mixture overnight at 37 ℃. The next day, the cells were washed with 4×SSC supplemented with 0.1% Tween 20 at 42 ℃ for 5 min, 2×SSC at 42 ℃ for 5 min, and 1×SSC at 42 ℃ for 5 min. Finally, cells were stained with 100 µl DAPI at room temperature for 10 min, washed with PBS for 5 min, and sealed. Images were captured by fluorescence confocal microscopy.

### Cell proliferation assays

Cell Counting Kit-8 (CCK-8) and colony formation assays were conducted to assess cell proliferation. In brief, for the CCK-8 assay, 2,000 TPC-1 and GLAG-66 cells per well were seeded in 96-well plates and incubated with 10 µl CCK-8 (Vazyme Biotech Co., Ltd.) per well for 1 h. The optical density was measured at 450 nm. For the colony formation assay, 1,000 TPC-1 and GLAG-66 cells were seeded in a 6-well plate, cultured for about 14 days, fixed with 4% paraformaldehyde at room temperature for 15 min, and stained with 1% crystal violet at room temperature for 5 min. Images were captured by Nikon Eclipse Ti2-U microscope and colonies containing > 50 cells were counted by image J software (version 1.5.3).

### Cell migration assays

Wound healing and Transwell assays were conducted to assess cell migration. For the wound healing assay, TPC-1 and GLAG-66 cells with > 80% confluence was scratched using a 200 µl pipette tip, and images of the scratches were captured by Nikon Eclipse Ti2-U microscope at 0 and 24 h and analyzed by Image J software (version 1.5.3). For the Transwell assay, 2–5 × 10^4^ TPC-1 and GLAG-66 cells in serum-free medium per 200 µl were added into the upper chamber and 600 µl complete media were added into the lower chamber (8 μm, Labselect; Beijing Labgic Technology Co., Ltd.). After 24 h of culture, cells were fixed with 4% paraformaldehyde at room temperature for 15 min, and stained with 1% crystal violet at room temperature for 5 min. Images were captured by Nikon Eclipse Ti2-U microscope and colonies were counted by image J software (version 1.5.3).

### RNA immunoprecipitation (RIP)

RIP was performed using the PureBinding RNA immunoprecipitation kit (Guangzhou Geneseed Biotech Co., Ltd). Briefly, 2 × 10^7^ TPC-1 cells were lysed with 1 ml Buffer A supplemented with 1% volume of protease inhibitor and 1% volume of RNase inhibitor for 10 min on ice, and vortexed twice for 5 s each time. Centrifuged at 14,000 ×g for 10 min at 4 °C. 200 µl protein A/G magnetic beads were incubated with either Ago2 antibody (5 µg; cat. no.67934-1-Ig, ProteinTech Group, Inc.) or mouse IgG (5 µg; cat.no. AC011, Abclonal) for 1 h at 4 ℃. Subsequently, the treated magnetic beads were incubated with cell lysate supernatant overnight at 4 ℃. The next day, 1 ml Buffer B was added to the magnetic bead mixture and vortexed for 2 min, placed on a magnetic rack to discard the supernatant, and repeated five times. 300 µl Buffer E was added to the magnetic bead mixture and vortexed for 30 s. The supernatant containing RNA was collected on a magnetic rack and was analyzed by RT-qPCR.

### Dual-luciferase reporter assay

The luciferase reporter plasmid pmirGLO, purchased from Suzhou GenePharma Co., Ltd., contained circLIFR wild-type (WT) and mutant (MUT) sequences, as well as TIMP2 WT and MUT sequences. TPC-1 cells were co-transfected with miR-429 negative control (NC)/mimic (50 nM) and WT/MUT (1 µg) at 37 °C for 8 h using Lipofectamine 3000 (Invitrogen; Thermo Fisher Scientific, Inc.). Luciferase activity was measured at 48 h post-transfection using the Dual-Luciferase Reporter Assay system (Promega Corporation). The firefly luciferase activity was normalized to Renilla luciferase activity and the results were shown as fold-change compared to mimic-NC.

### Western blotting

Protein samples were obtained by lysis of TPC-1 and GLAG-66 cells with RIPA lysis buffer (Beyotime Institute of Biotechnology) supplemented with PMSF and phosphatase inhibitors (Biosharp Life Sciences). The protein concentration was determined by the BCA Protein Assay Kit (Beyotime Institute of Biotechnology). Proteins (25 µg/ lane) were separated by SDS-PAGE on a 12% gel and transferred to PVDF membranes (MilliporeSigma). Membranes were blocked with 5% skim milk for 1 h at room temperature and incubated with primary antibodies TIMP2 (1:500; cat.no. WL01209, Wanlei Biotechnology Co., Ltd.), GAPDH (1:50000; cat. no. 60004-1-Ig, Proteintech Group, Inc.) overnight at 4 °C, and washed 3 times for 10 min with TBS containing 0.1% Tween-20 (Sinopharm Chemical Reagent Co., Ltd.) at room temperature. Membranes were incubated with secondary antibodies HRP-conjugated goat anti-rabbit IgG (H&L) (1:2000, cat.no. CW0103, Cowin Biotech Co., Ltd.) or HRP-conjugated goat anti-mouse IgG (H&L) (1:2000, cat.no. CW0102, Cowin Biotech Co., Ltd.) for 1 h at room temperature and washed 3 times for 10 min with TBST containing 0.1% Tween-20 (Sinopharm Chemical Reagent Co., Ltd.) at room temperature. Protein bands were visualized using ECL substrates (MilliporeSigma) on the ImageQuant LAS 4000 Mini imager (General Electric Company). Bands were analyzed using Image J software (version 1.5.3).

### Xenograft tumor model

Eight four-week-old female BALB/c nude mice were purchased from Changzhou Cavens Experimental Animal Co., Ltd. and kept in a controlled environment with a 12-hour light/dark cycle, a temperature of 22 °C, and 60% relative humidity. The mice were kept free of pathogens. The mice were divided into two groups at random, with four mice per group. The right back flank of BALB/c nude mice was injected with a total of 5 × 10^6^ GLAG-66 cells in 100 µl stably transfected with overexpression (OE)-circLIFR or Vector. The maximum tumor volume was ≤ 1,000 mm^3^. Every day the tumor’s growth was observed, and its volume was computed using the following formula: volume = 0.5 × length × width^2^. The mice were euthanized four weeks after implantation by intraperitoneal injection of sodium pentobarbital (150 mg/kg) to conduct further analysis. The Institutional Animal Care and Use Committee (IACUC) of Jiangsu University provided approval for this research, and all aspects of its conduct, including the care and use of animals, were carried out strictly in compliance with its rules (Approval No.: UJS-IACUC-2,023,063,003).

### Immunohistochemistry (IHC)

Tumor tissues treated in 4% paraformaldehyde were embedded in paraffin and sectioned into 5 μm-thick slices. Following a 10 min xylene soak, the slices were taken out of the solvent and soaked twice in anhydrous ethanol for 3 min each, followed by 3 min each in 95%, 85%, 70%, and H_2_O ethanol. Slices were cleaned with PBS, then submerged in a citric acid antigen repair solution (pH 6.0) and boiled for 6 min each time in water. The slices were cooled, and then immersed in PBS solution three times for 5 min each. To stop endogenous peroxidase activity, 3% H_2_O_2_ was added to the slices and let them sit at room temperature for 10 min. Slices were incubated at 4 °C overnight with Ki-67 (1:300; cat. no. GB111141-100, Wuhan Servicebio Technology Co., Ltd.) and TIMP2 (1:200; cat. no. WL01209, Wanlei Biotechnology Co., Ltd.) antibodies. The secondary antibody was incubated for 30 min at room temperature. Slices were restained with hematoxylin for 30 s after being treated with DAB (Wuhan Servicebio Technology Co., Ltd.) for 5 min. PANNORAMIC MIDI II slide scanner was used to take pictures (3DHISTECH Ltd.).

### Statistical analysis

Data analysis and plotting were performed using GraphPad Prism 8. Data are presented as the mean ± SD. Experiments were replicated at least thrice. Student’s t-test was used to analyze differences among groups. Pearson correlation analysis was also performed. *P* < 0.05 was considered to indicate a statistically significant difference.

## Results

### CircLIFR is downregulated in PTC

**Fig. 1:**
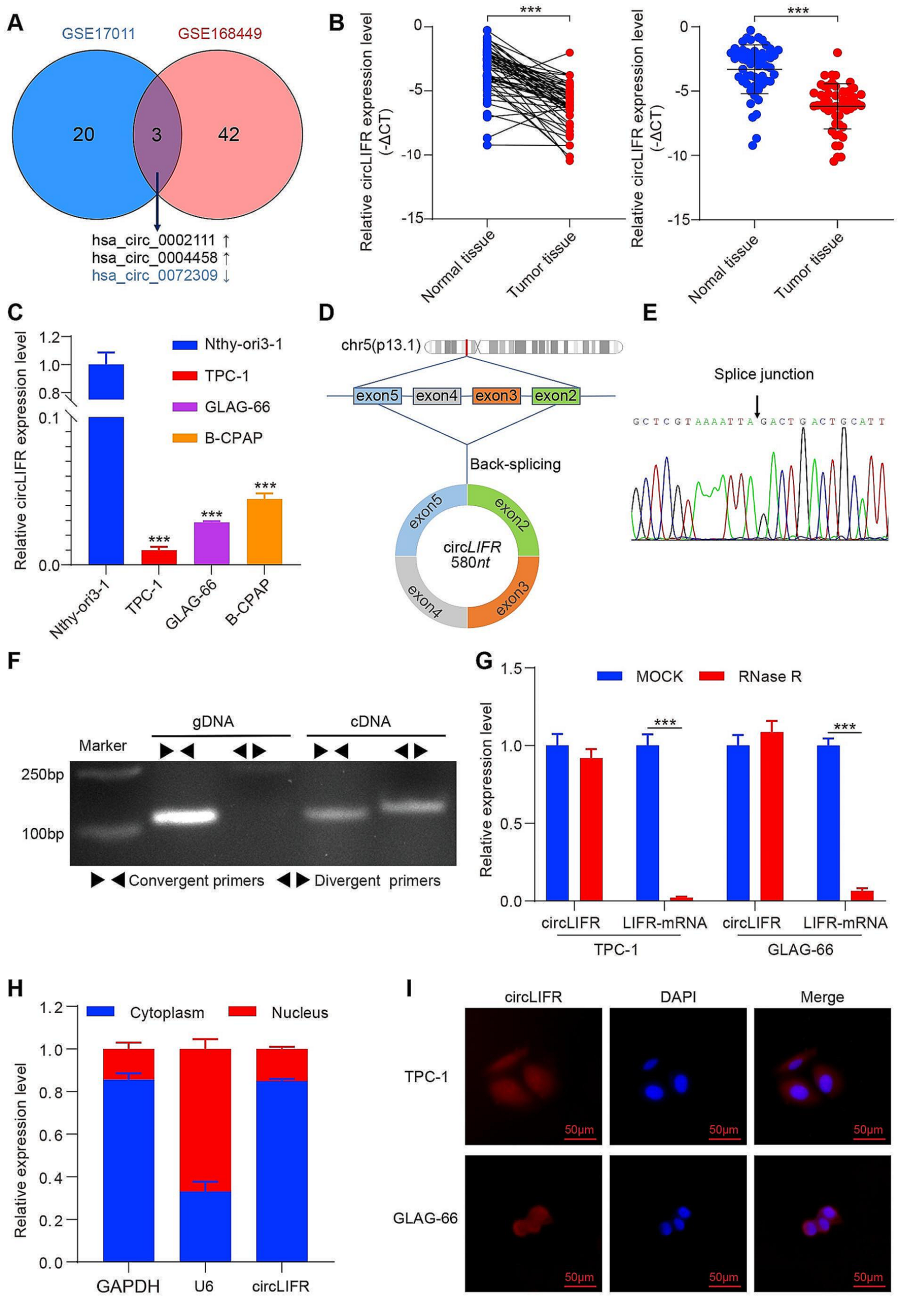
CircLIFR is down-regulated in PTC. **(A)** Differentially expressed circRNAs were analyzed using the GEO database. The common circRNAs were listed. **(B)** Relative expression of circLIFR in tumor tissues and normal tissues of patients with PTC was detected by RT-qPCR (*n* = 53). **(C)** Relative expression of circLIFR in cell lines was detected by RT-qPCR. **(D)** Genomic location of circLIFR. CircLIFR was formed by the back-splicing of exons 2–5 of LIFR. **(E)** The back splice junction site of circLIFR was identified by Sanger sequencing. **(F)** PCR assay of gDNA and cDNA using divergent and convergent primers of circLIFR. **(G)** The stability of circLIFR and LIFR mRNA was detected by RNase R. (H) Subcellular localization of circLIFR was analyzed by RNA nucleocytoplasmic separation. (I) Subcellular localization of circLIFR was analyzed by FISH. Scale bar = 50 μm. ***P < 0.001

To identify circRNAs with pivotal roles in PTC, the Bioinformatics analyzed the GSE171011 and GSE168449 datasets from the GEO database. This analysis revealed three commonly differentially expressed circRNAs (Fig. 1A). Previous studies have documented the oncogenic effects of hsa_circ_0002111 and hsa_circ_0004458 in PTC (Du et al. 2021; Jin et al. 2018). However, to the best of our knowledge, the function and mechanism of hsa_circ_0072309, also referred to as circLIFR, in PTC remain elusive.

Bioinformatics analysis demonstrated marked downregulation of circLIFR in PTC tissues. Using RT-qPCR, 53 pairs of PTC and adjacent normal tissues were examined. The results indicated decreased circLIFR expression in PTC tissues compared with normal tissues (Fig. 1B). This observation was verified in PTC cell lines (TPC-1, GLAG-66, and B-CPAP), in which circLIFR levels were notably lower than those in Nthy-ori3-1 cells (Fig. 1C). Based on these findings, circLIFR was selected for further analysis.

CircLIFR is a 580-nucleotide circRNA originating from the back splicing of exon 2–5 of the LIFR gene located on chromosome 5 (Fig. 1D), and was validated through Sanger sequencing, affirming the presence of back splicing junctions in PCR-amplified products using different primers (Fig. 1E). Furthermore, PCR electrophoresis analysis demonstrated the template for PCR amplification of divergent primers was cDNA, not genomic DNA. (Fig. 1F). After RNase R treatment of total RNA, the results revealed a marked reduction in linear LIFR mRNA abundance, contrasting with the relatively unchanged levels of circLIFR (Fig. 1G). RNA nucleocytoplasmic separation indicated the presence of circLIFR in both the cytoplasm and nucleus (Fig. 1H), and this finding was further validated by FISH (Fig. 1I). Collectively, these findings suggested that circLIFR was downregulated in PTC and could be associated with the disease progression.

### CircLIFR inhibits PTC cell proliferation and migration

**Fig. 2:**
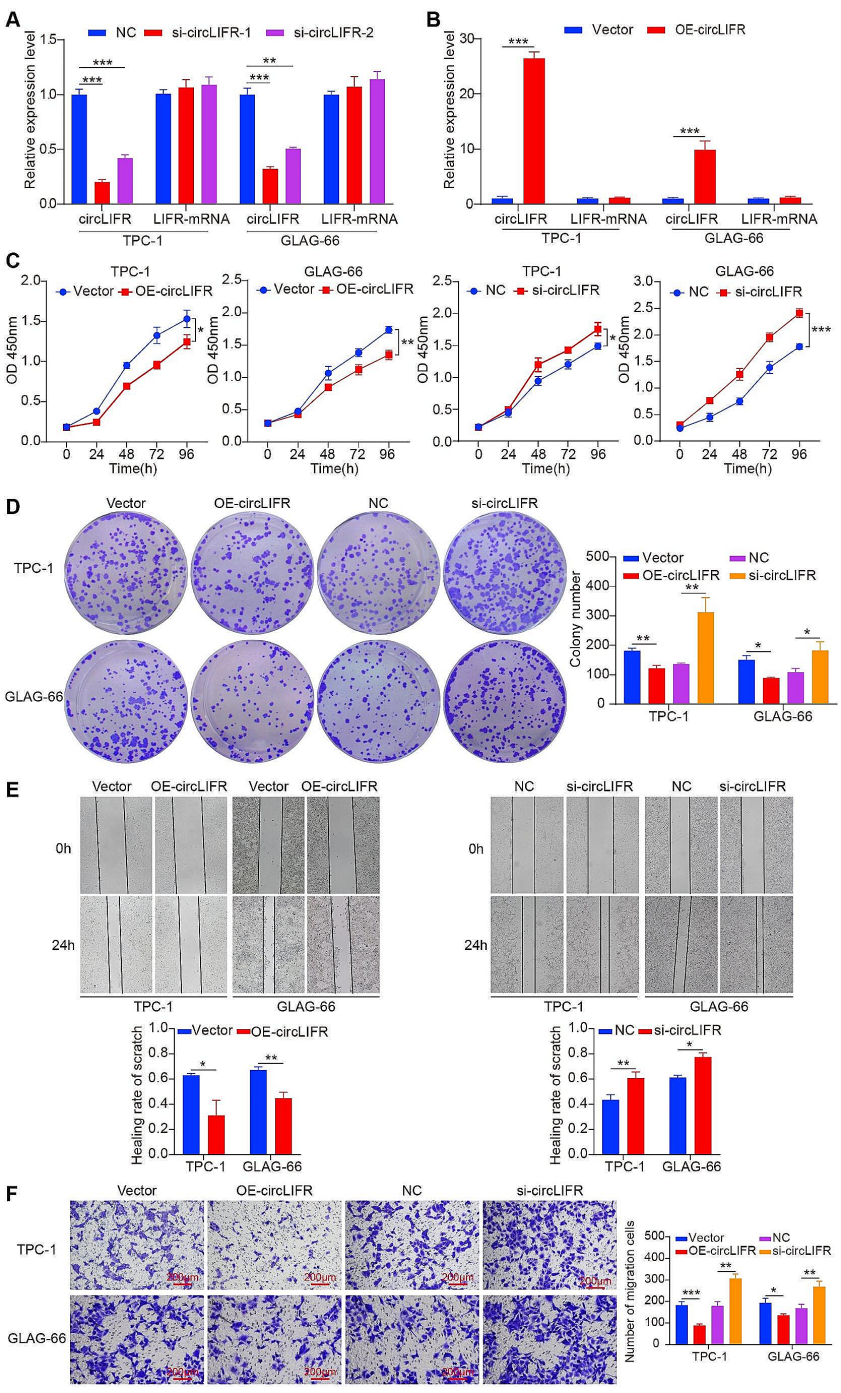
CircLIFR inhibits the proliferation and migration of PTC in vitro. **(A)** and **(B)** RT-qPCR analysis of siRNA transfection efficiency (A) or lentivirus overexpression efficiency (B) in PTC cells. **(C)** and **(D)** CCK-8 assays (C) and colony formation assays (D) were performed to determine the ability of proliferation in PTC cells transfected with si-circLIFR or NC and transfected with OE-circLIFR or Vector. **(E)** and **(F)** Cell migratory capabilities were evaluated by wound healing assays (E) and Transwell assays (F) in PTC cells transfected with si-circLIFR or NC and transfected with OE-circLIFR or Vector. Scale bar = 200 μm. *P < 0.05, **P < 0.01, ***P < 0.001

To elucidate the function of circLIFR in PTC cells, two siRNAs targeting its back-splicing junction were designed and transfected into TPC-1 and GLAG-66 cells. si-circLIFR suppressed circLIFR expression without affecting LIFR mRNA (Fig. 2A). For further experiments, si-circLIFR-1 was selected due to its superior knockdown efficiency. Stably transfected cell lines were established using lentivirus, increasing circLIFR expression in TPC-1 and GLAG-66 cells without affecting LIFR mRNA (Fig. 2B). Subsequently, CCK-8 and colony formation assays were conducted to assess cell proliferation. Both assays indicated that circLIFR knockdown amplified proliferation in TPC-1 and GLAG-66 cells, whereas its overexpression had the opposite effect (Fig. 2C and D). The present study also assessed cell migration using wound healing and Transwell assays. These assays revealed enhanced migration in TPC-1 and GLAG-66 cells with circLIFR knockdown, while its overexpression inhibited migration (Fig. 2E and F). Collectively, these findings suggested the potential of circLIFR to suppress PTC cell proliferation and migration.

### CircLIFR serves as a miR-429 sponge in PTC

**Fig. 3:**
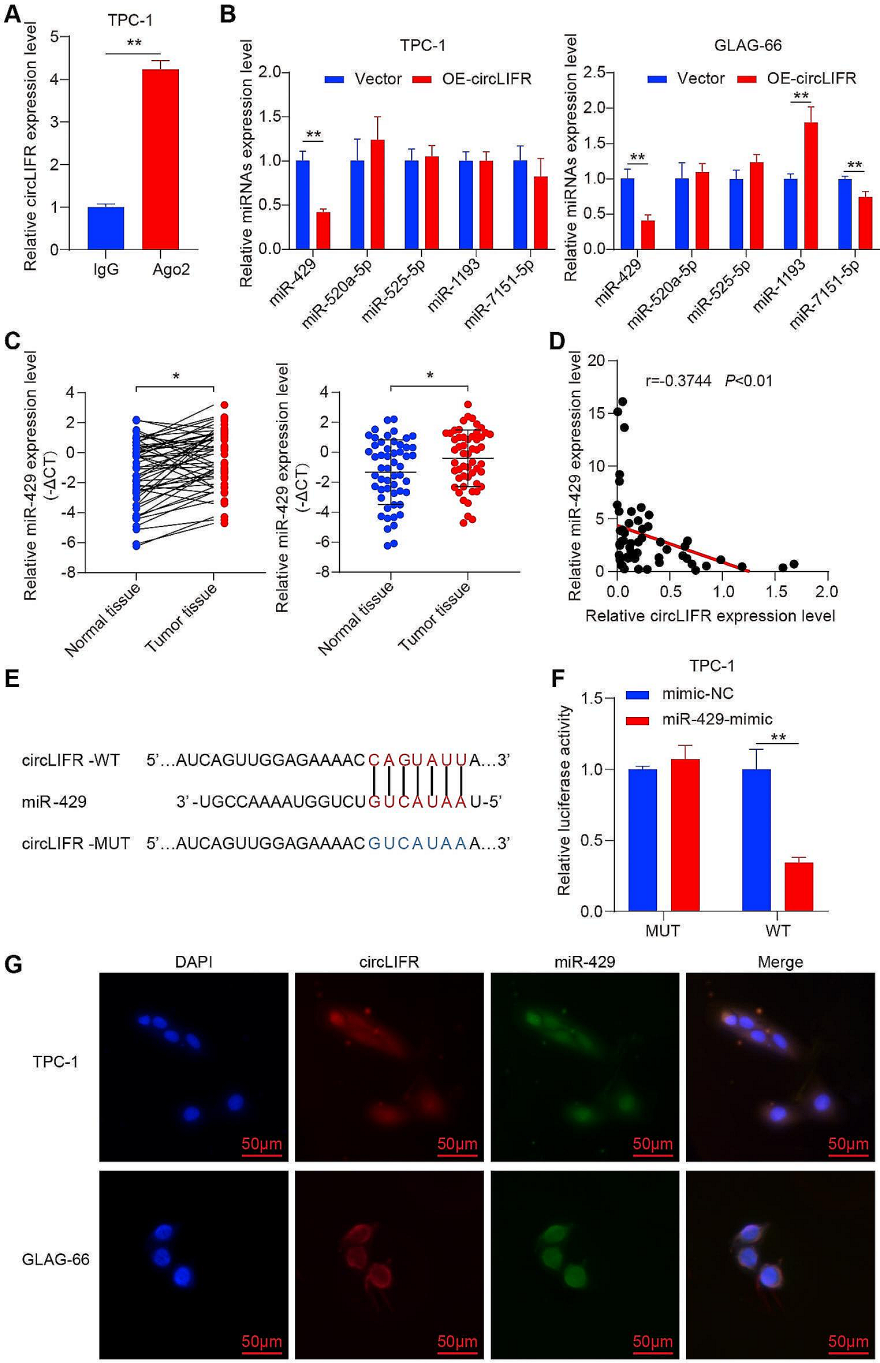
CircLIFR acts as a sponge for miR-429 in PTC. **(A)** RIP assay was applied using Ago2 antibody in PTC cells. The relative expression of circLIFR was detected by RT-qPCR. **(B)** The relative expression of candidate miRNAs was detected by RT-qPCR in PTC cells. **(C)** RT-qPCR analysis for the expression of miR-429 in PTC tissues. **(D)** Pearson correlation analysis of circLIFR and miR-429 expression in tumor tissues of patients with PTC. **(E)** Schematic illustration of circLIFR-WT and circLIFR-MUT luciferase reporter vectors. **(F)** Relative luciferase activities were detected in TPC-1 cells after co-transfection with circLIFR-WT or circLIFR-MUT and miR-429-mimic or NC. **(G)** FISH for the co-localization of miR-429 (green) and circLIFR (red) in PTC cells. Scale bar = 50 μm. *P < 0.05, **P < 0.01

RNA immunoprecipitation assays were conducted to assess the binding potential between circLIFR and miRNAs. The results indicated that the Ago2 group exhibited a substantial enrichment in circLIFR compared with the IgG group (Fig. 3A). Using the ENCORI database, 11 potential miRNAs interacting with circLIFR were identified. Among these, five (miR-429, miR-520a-5p, miR-525-5p, miR-1193, and miR-7151-5p) were shortlisted based on prediction scores (Table S4). The present study then assessed the impact of circLIFR on miRNAs using RT-qPCR. The results demonstrated that miR-429 expression was markedly decreased in TPC-1 and GLAG-66 cells overexpressing circLIFR (Fig. 3B). Additionally, analysis of miR-429 expression in 53 paired PTC and normal tissues revealed a marked increase in miR-429 expression in PTC tissues (Fig. 3C). Pearson correlation analysis further indicated a negative correlation between circLIFR and miR-429 (Fig. 3D). A dual luciferase reporter vector was constructed to confirm direct binding between circLIFR and miR-429 (Fig. 3E). Co-transfection of circLIFR WT or MUT plasmid with mimic-NC or miR-429 mimic into TPC-1 cells indicated a marked reduction in luciferase activity in the miR-429 mimic group (Fig. 3F). RNA FISH was further performed, and this revealed the co-localization of circLIFR and miR-429 in both the cytoplasm and nucleus (Fig. 3G). These collective findings established the binding interaction between circLIFR and miR-429, positioning circLIFR as a sponge for miR-429 and highlighting its functional role in the context of PTC.

### miR-429 promotes PTC cell proliferation and migration

**Fig. 4:**
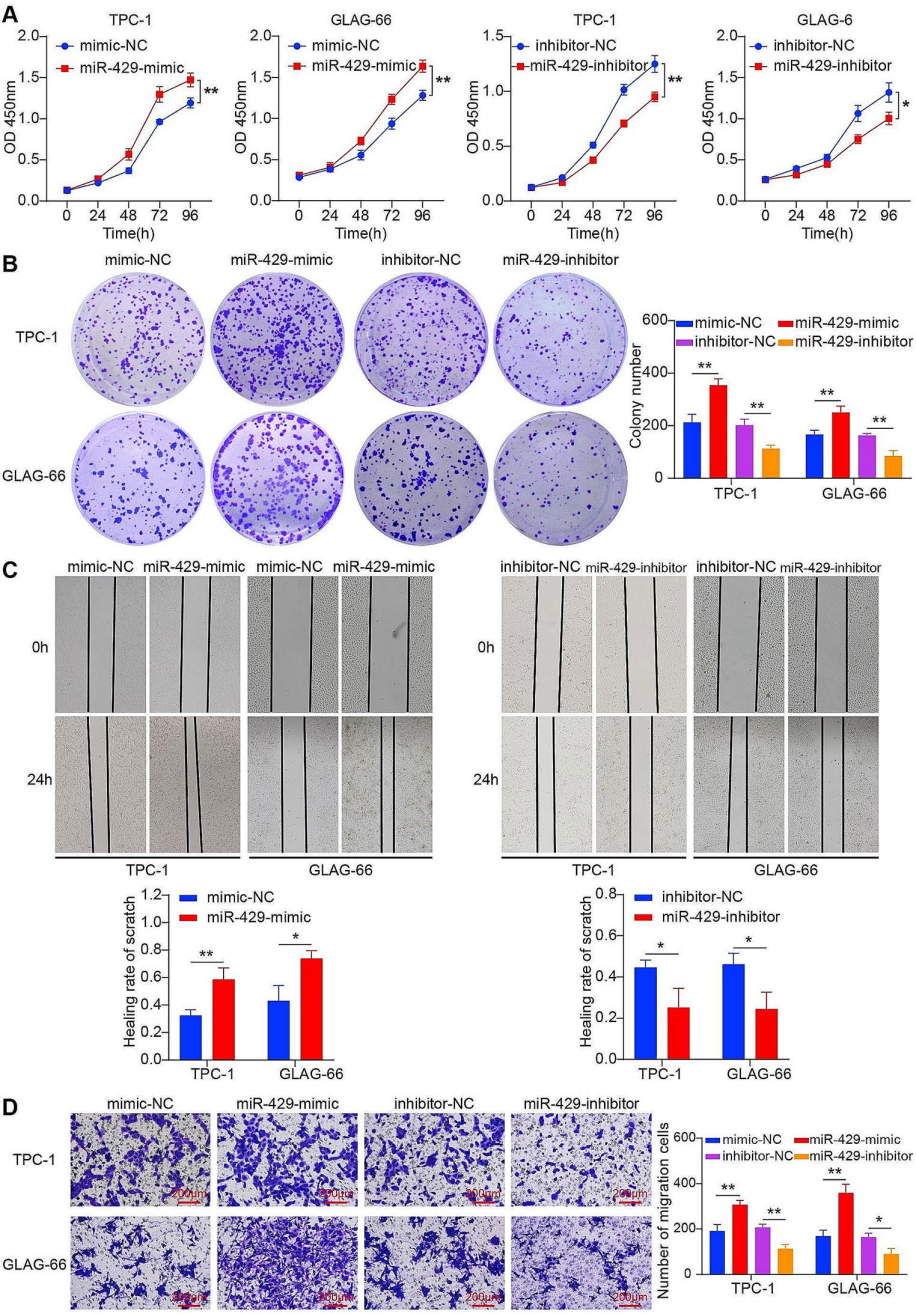
miR-429 promotes PTC cell proliferation and migration. **(A)** and **(B)** CCK-8 assays (A) and colony formation assays (B) were performed to determine the ability of proliferation in PTC cells transfected with miR-429 mimic or inhibitor and corresponding negative control. **(C)** and **(D)** Cell migratory capabilities were evaluated by wound healing assays (C) and Transwell assays (D) in PTC cells transfected with miR-429 mimic or inhibitor and corresponding negative control. Scale bar = 200 μm. *P < 0.05, **P < 0.01

The oncogenic role of miR-429 was assessed in PTC cells to measure cell proliferation and migration. Cell proliferation was measured using the Cell Counting Kit-8 (CCK-8) and colony formation assays. PTC cells transfected with a miR-429 mimic showed a significant increase in proliferation compared to negative control (NC) group (Fig. 4A and B). In contrast, inhibited miR-429 showed a marked decrease in proliferation, underlining the proliferative effect of miR-429. The role of miR-429 in cell migration was investigated using wound healing and Transwell migration assays. In comparison to the NC group, the miR-429 mimic significantly increased PTC cells’ migration and proliferation (Fig. 4C and D). Conversely, inhibition of miR-429 resulted in a significant decrease in cell migration, demonstrating a vital role of miR-429 in aiding both the proliferation and migration of PTC.

### miR-429 targets TIMP2

**Fig. 5:**
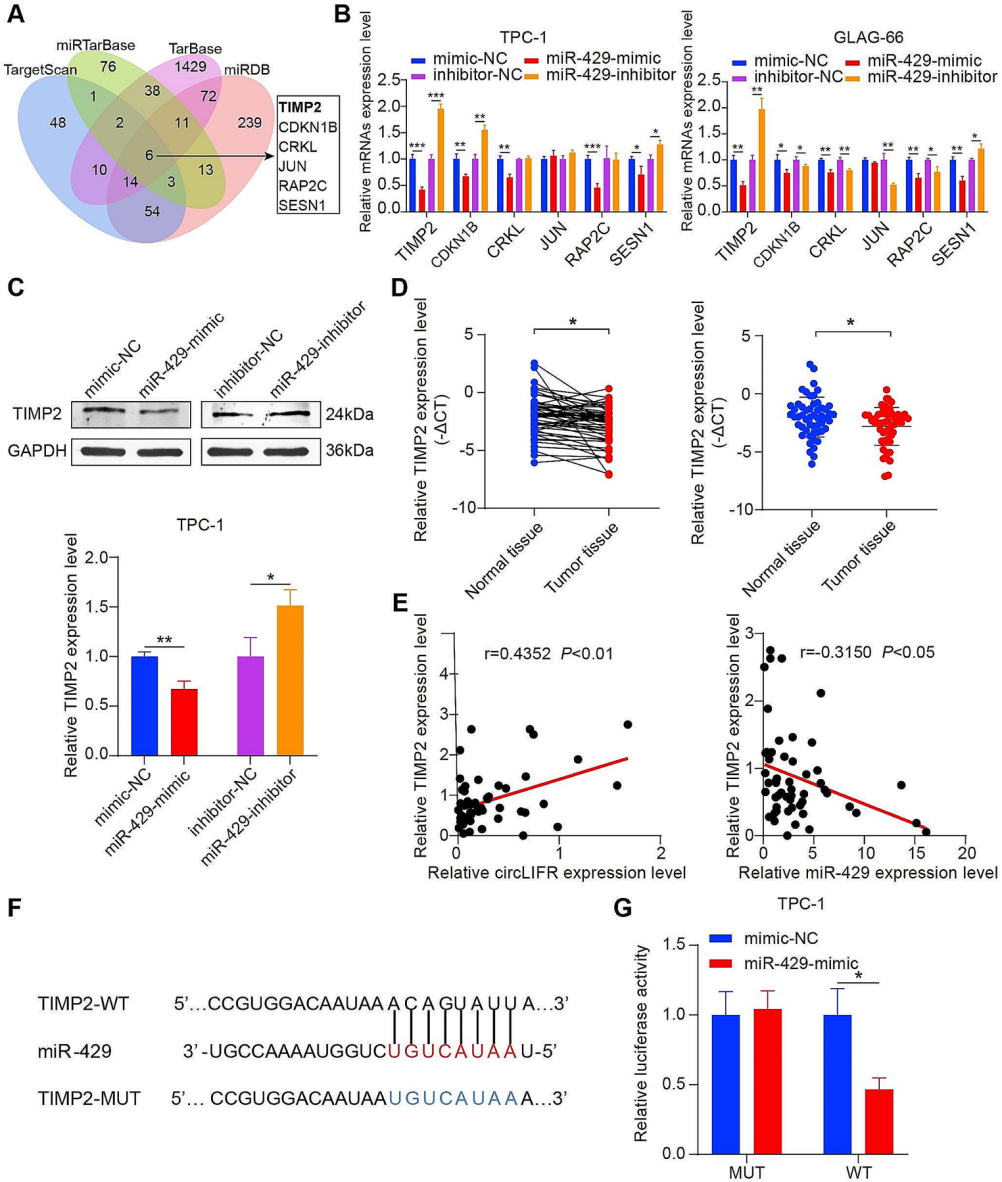
miR-429 targets TIMP2. **(A)** Schematic illustration exhibiting overlapping of the target genes of miR-429 predicted by miRDB, miRTarBase, TarBase, and TargetScan. **(B)** The mRNA expression of potential target genes for miR-429 in PTC cells transfected with miR-429 mimic or inhibitor was detected by RT-qPCR. **(C)** Relative protein levels of TIMP2 were evaluated by western blotting in TPC-1 cells transfected with the miR-429 mimic or inhibitor. **(D)** RT-qPCR analysis for the expression of TIMP2 in PTC tissues. **(E)** Pearson correlation analysis of TIMP2 and miR-429 or circLIFR expression in tumor tissues of patients with PTC. **(F)** Schematic illustration of TIMP2-WT and TIMP2-MUT luciferase reporter vectors. **(G)** Relative luciferase activities were detected in TPC-1 cells after co-transfection with TIMP2-WT or TIMP2-MUT and miR-429-mimic or NC. *P < 0.05, **P < 0.01, ***P < 0.001

To determine the downstream target genes of miR-429, the TargetScan, miRDB, TarBase, and miRTarBase databases were utilized. This analysis identified 6 common genes (Fig. 5A). Among them, TIMP2 showed the most distinct changes following transfection with either miR-429 mimic or inhibitor (Fig. 5B). Western blotting confirmed that TIMP2 protein levels were decreased following transfection with miR-429 mimic and increased following transfection with its inhibitor (Fig. 5C). Further investigation using RT-qPCR revealed lower TIMP2 expression in PTC tissues than in normal tissues (Fig. 5D). Pearson correlation analysis indicated a negative correlation between TIMP2 and miR-429, and a positive correlation between TIMP2 and circLIFR (Fig. 5E). Dual luciferase reporter vectors were constructed and co-transfected with mimic-NC or miR-429 mimic to validate the binding of miR-429 to TIMP2 (Fig. 5F). The miR-429 mimic group exhibited markedly reduced luciferase activity, further confirming the binding interaction (Fig. 5G). Collectively, these results established TIMP2 as a target gene of miR-429.

### CircLIFR suppresses PTC cell proliferation and migration via the circLIFR/miR-429/TIMP2 axis

**Fig. 6:**
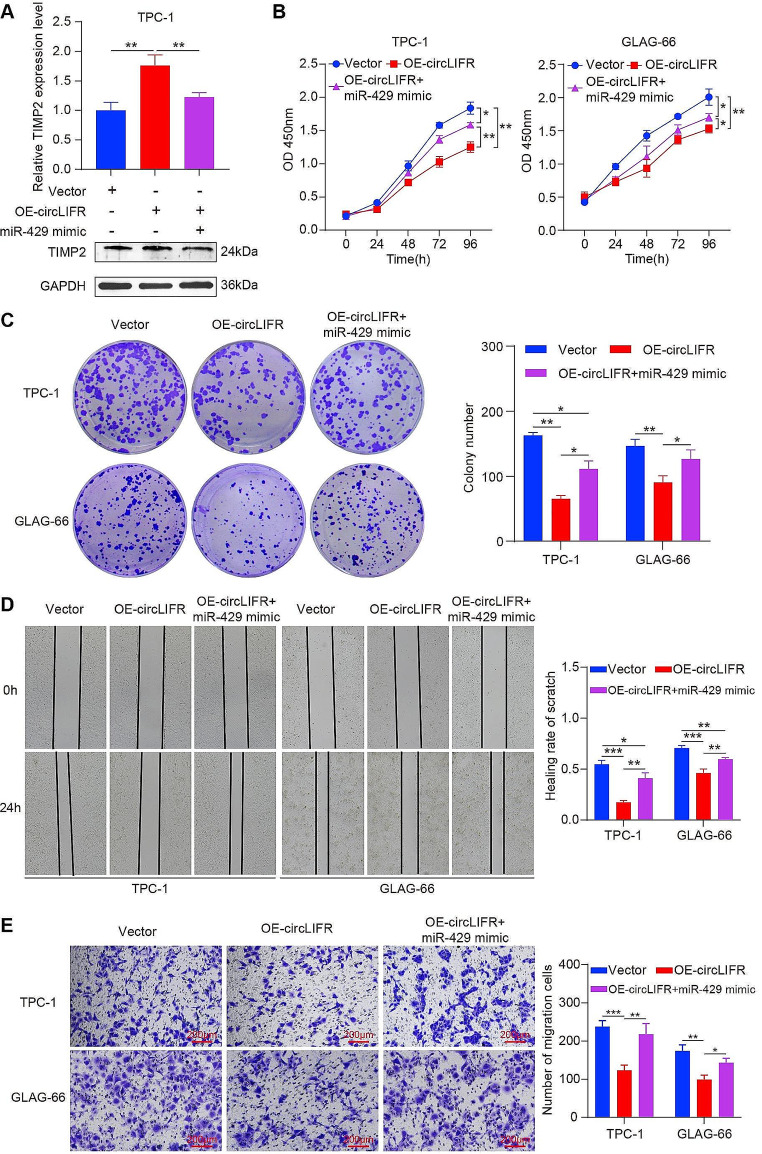
CircLIFR suppresses PTC cell proliferation and migration through the circLIFR/miR-429/TIMP2 axis. (A) Relative protein levels of TIMP2 were detected in TPC-1 cells transfected with Vector, OE-circLIFR, and mimic by western blotting. (B) and (C) CCK-8 assays (B) and colony formation assays (C) were performed to determine the ability of proliferation in PTC cells transfected with miR-429 mimic and Vector or OE-circLIFR. (D) and (E) Cell migratory capabilities were evaluated by wound healing assays (D) and Transwell assays (E) in PTC cells transfected with miR-429 mimic and Vector or OE-circLIFR. Scale bar = 200 μm. *P < 0.05, **P < 0.01, ***P < 0.001

To explore the interactions among circLIFR, miR-429, and TIMP2, rescue experiments were conducted. Co-transfection of overexpression (OE)-circLIFR and miR-429 mimic into cells revealed reduced TIMP2 expression in OE-circLIFR cells. However, this reduction was reversed when OE-circLIFR was co-transfected with miR-429 mimic (Fig. 6A). Furthermore, circLIFR overexpression inhibited PTC cell proliferation and migration, and these effects were mitigated upon co-transfection with miR-429 mimic (Fig. 6B-E). These findings suggested that circLIFR modulated TIMP2 by acting as a miR-429 sponge, subsequently inhibiting PTC cell proliferation and migration.

### Overexpression of circLIFR suppresses tumor growth in vivo

**Fig. 7:**
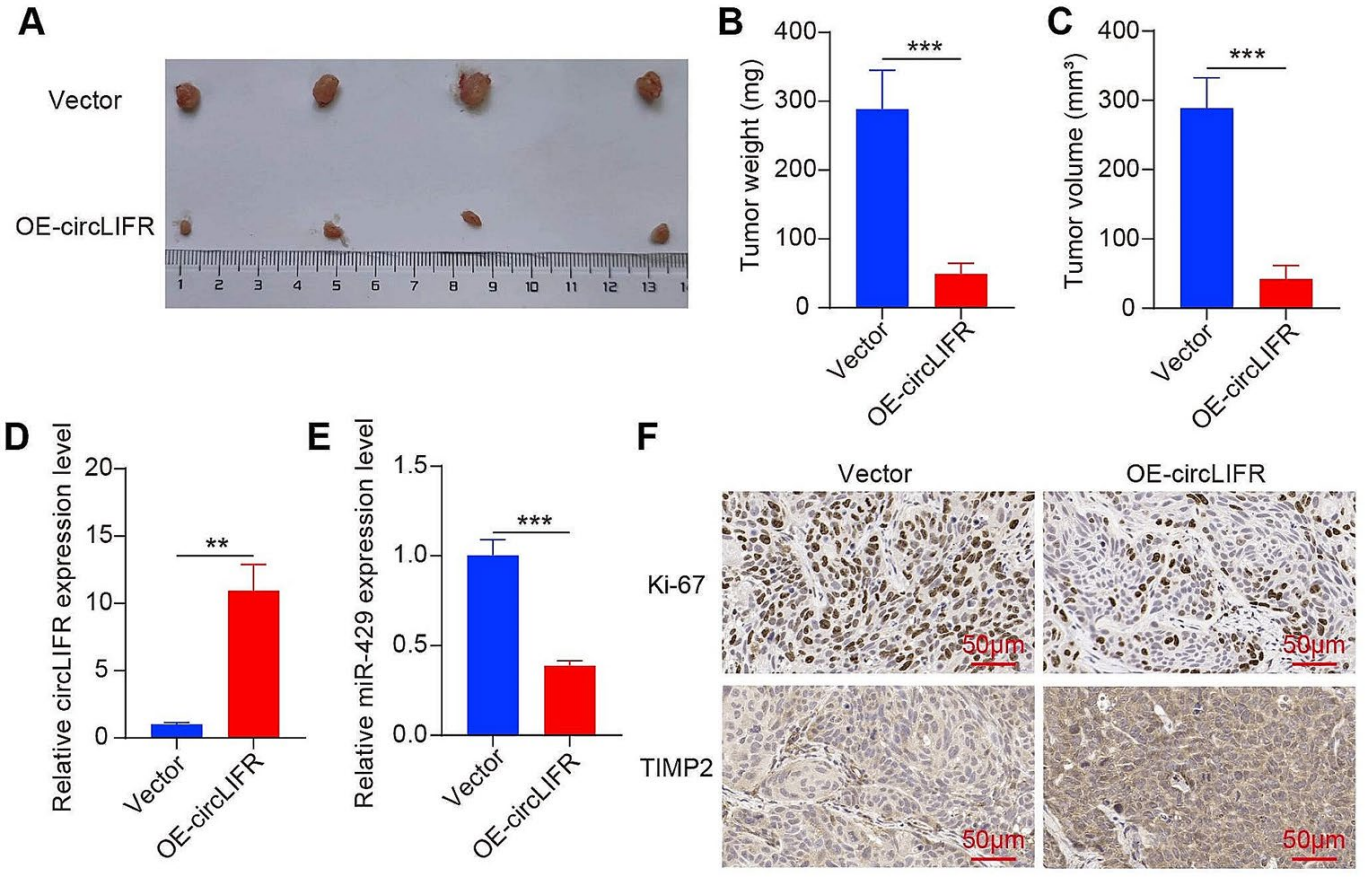
Overexpression of circLIFR suppresses tumor growth in vivo. (A) images of xenograft tumors. (B) Tumor weight. (C) Tumor volume. (D) RT-qPCR analysis of the relative expression levels of circLIFR in tumor tissues. (E) RT-qPCR analysis of the relative expression levels of circLIFR in tumor tissues. (F) IHC analysis of expression levels of Ki-67 and TIMP2 proteins in tumor tissues. Scale bar = 100 μm. **P < 0.01, ***P < 0.001

The Xenograft tumor model was established to investigate the in vivo function of circLIFR. Our results revealed that the tumor volume and weight were significantly reduced in the circLIFR overexpression group compared to the vector group after tumor formation in nude mice (Fig. 7A-C). RT-qPCR analysis demonstrated a significant increase in circLIFR expression in tumor tissues with circLIFR overexpression, while miR-429 expression levels were decreased (Fig. 7D and E). Immunohistochemical analysis confirmed that circLIFR overexpression led to a decrease in Ki-67 expression and an increase in TIMP2 expression within the tumor tissues (Fig. 7F). Therefore, our findings indicate that overexpressing circLIFR effectively suppressed tumor growth in vivo.

## Discussion

PTC is the predominant endocrine tumor and has become a significant health concern (Chen et al. 2023). Previous evidence has highlighted the pivotal role of circRNA in the pathogenesis of various ailments, including cancer (Liu et al. 2019, 2023a, b; Huang et al. 2023; Chen 2020). Our study is the first to demonstrate the tumor-suppressive role of circLIFR in PTC. We found that overexpression of circLIFR inhibited PTC cell proliferation and migration, while knockdown of circLIFR had the opposite effect. Mechanistically, circLIFR functions as a sponge for miR-429, leading to the upregulation of TIMP2, which in turn inhibits PTC cell proliferation and migration (Fig. S1). This novel mechanism underscores the potential of circLIFR as a tumor suppressor and a promising therapeutic target in PTC. Our findings not only enhance the understanding of circRNA functions in cancer but also offer new insights into PTC pathogenesis and potential treatments.

The mechanisms through which circRNAs influence pathophysiological processes in cancer are complex, encompassing miRNA sponging, RNA-binding proteins (RBPs) interactions, and peptide translation (Li et al. 2020; Chen 2020; Panda 2018). The most extensively researched is the competing endogenous RNA (ceRNA) mechanism, where circRNAs sponge miRNAs, preventing miRNA-mRNA 3’UTR interactions (Lei et al. 2020). For instance, circ_0001018 binds miR-338-3p, enhancing SOX4 expression and thereby augmenting PTC cell invasion (Luo et al. 2021). Similarly, circLIFR has been identified as a tumor suppressor in hepatocellular carcinoma by modulating the miR-624-5p and GSK-3β/β-catenin signaling pathway (Yang et al. 2022). The present findings resonate with these studies, reinforcing the potential tumor-suppressive role of circLIFR in PTC. However, it is crucial to note that not all circRNAs serve as miRNA sponges; their cytoplasmic location is a prerequisite for this function (Misir et al. 2022; Zhang et al. 2019). A recent study, contrasting the present findings, has suggested that circLIFR is primarily located in the nucleus, interacting with MSH2 to modulate cisplatin sensitivity in bladder cancer via the MutSα/-p73 axis (Zhang et al. 2021a, b). These discrepancies might arise from the varied subcellular localization of circLIFR across different cell lines.

miRNAs are integral to the ceRNA network and are dysregulated in numerous types of cancer. The role of miR-429 as either an oncogene or tumor suppressor has been documented across various types of cancer (Klicka et al. 2022). For instance, in hepatocellular carcinoma, miR-429 targets CRKL, inhibiting tumor migration and invasion via the Raf/MEK/ERK signaling pathway and epithelial-mesenchymal transition (Guo et al. 2018). Elevated miR-429 levels in HER2 + breast cancer (BC) enhance BC cell proliferation and migration (Cava et al. 2020). Furthermore, in colorectal cancer tissues, miR-429 overexpression, by directly targeting SOX2 in HT-2 cells, impedes cell apoptosis (Li et al. 2013). The present data revealed the upregulation of miR-429 in PTC tissues and cells. Functional assays further demonstrated the role of miR-429 in promoting PTC proliferation and migration. miR-429 overexpression weakened the tumor-suppressive effects of circLIFR on PTC cells. The dual luciferase reporter assay corroborated the binding affinity between miR-429 and circLIFR, suggesting a regulatory role of circLIFR in PTC progression via miR-429 binding.

TIMP2 is a tissue inhibitor of MMPs family members and acts as a specific matrix metalloproteinase inhibitor (Stetler-Stevenson 2008). It serves a role in diverse pathophysiological processes, including cell growth, differentiation, apoptosis, angiogenesis, migration, and invasion (Stetler-Stevenson 2008; Peeney et al. 2022; Escalona et al. 2020). We found that TIMP2 expression was downregulated in PTC tissues, and TIMP2 expression decreased or increased following overexpression or inhibition of miR-429, respectively. The dual luciferase reporter assay further confirmed the direct binding between miR-429 and TIMP2. Therefore, this study identifies TIMP2 as a downstream target of miR-429. By obstructing the miR-429 and TIMP2 interaction, circLIFR elevated TIMP2 expression, consequently diminishing PTC cell proliferation and migration.

However, this study has some limitations. CircRNAs have been shown to be stably detectable in plasma, indicating their potential as tumor biomarkers and therapeutic targets (Zhang et al. 2021a, b). Therefore, further investigation is required to determine whether circLIFR can also be consistently detected in plasma. Additionally, it is necessary to explore the correlation between circLIFR expression and clinical stages as well as other pathological features. Such studies will be essential for understanding the clinical relevance and potential applications of circLIFR in PTC.

## Conclusion

In summary, the present study revealed the inhibitory role of circLIFR in PTC progression. CircLIFR was downregulated in PTC tissues and cells and inhibited the proliferation and migration of PTC cells, which was mediated via the miR-429/TIMP2 axis. Consequently, our study provides a biomarker and potential therapeutic target for PTC.

## Electronic supplementary material

Below is the link to the electronic supplementary material.

Supplementary Material 1

Supplementary Material 2

## Data Availability

No datasets were generated or analysed during the current study.
