# Supplementary material for: Circular RNA circLIFR suppresses papillary thyroid cancer progression by modulating the miR-429/TIMP2 axis
Source: J Cancer Res Clin Oncol. 2024 Jun 25;150(6):323. doi: 10.1007/s00432-024-05839-7 (PMC11196293; doi:10.1007/s00432-024-05839-7)
Supplement: Supplementary file 1 — Supplementary Material 1 [file 432_2024_5839_MOESM1_ESM.docx]

Supplementary Material

**Table S1** Sequences for RNA oligo for transfection

| **RNA oligo for transfection** | | |
| --- | --- | --- |
| **Name** | **Sequence** | **Source** |
| siRNA NC | Sense 5′-UUCUCCGAACGUGUCACGUTT-3′  Antisense 5′-ACGUGACACGUUCGGAGAATT-3′ | Suzhou GenePharma Co., Ltd |
| si-circLIFR-1 | Sense 5′-CUCGUAAAAUUAGACUGACTT-3′  Antisense 5′-GUCAGUCUAAUUUUACGAGTT-3′ | Suzhou GenePharma Co., Ltd |
| si-circLIFR-2 | Sense 5′-GUAAAAUUAGACUGACUGCTT-3′  Antisense 5′-GCAGUCAGUCUAAUUUUACTT-3′ | Suzhou GenePharma Co., Ltd |
| mimic NC | Sense 5′-UUGUACUACACAAAAGUACUG-3′  Antisense 5′-GUACUUUUGUGUAGUACAAUU-3′ | Suzhou GenePharma Co., Ltd |
| miR-429 mimic | Sense 5′-UAAUACUGUCUGGUAAAACCGU-3′  Antisense 5′-GGUUUUACCAGACAGUAUUAUU-3′ | Suzhou GenePharma Co., Ltd |
| inhibitor NC | 5′-CAGUACUUUUGUGUAGUACAA-3′ | Suzhou GenePharma Co., Ltd |
| miR-429 inhibitor | 5′-ACGGUUUUACCAGACAGUAUUA-3′ | Suzhou GenePharma Co., Ltd |

**Table S2** Primer sequences for qPCR

| **Primer sequences for qPCR** | | |
| --- | --- | --- |
| **Name** | **Sequence** | **Source** |
| circLIFR | F 5′-TCCACACCGCTCAAATGTTA-3′  R 5′-ATCCAGGATGGTCGTTTCAA-3′ | Sangon Biotech Co., Ltd |
| GAPDH | F 5′-AGAAGGCTGGGGCTCATTTG-3′  R 5′-AGGGGCCATCCACAGTCTTC-3′ | Sangon Biotech Co., Ltd |
| U6 | F 5′-CTCGCTTCGGCAGCACA-3′  R 5′-AACGCTTCACGAATTTGCGT-3′ | Sangon Biotech Co., Ltd |
| c-circLIFR | F 5′-TGGAAAGCACCCTCTGGAAC-3′  R 5′-TCACCATGTGAAAGAGCTGGAA-3′ | Sangon Biotech Co., Ltd |
| LIFR-mRNA | F 5′-GCTGTCATTGTTGGAGTGGTG-3′  R 5′-CTGCTTCCCTCACAGACACT-3′ | Sangon Biotech Co., Ltd |
| miR-429 | F 5′-CGCGCGTAATACTGTCTGGTAA-3′  R 5′-AGTGCAGGGTCCGAGGTATT-3′ | Sangon Biotech Co., Ltd |
| miR-520a-5p | F 5′-CGCGCTCCAGAGGGAAGTA-3′  R 5′-AGTGCAGGGTCCGAGGTATT-3′ | Sangon Biotech Co., Ltd |
| miR-525-5p | F 5′-GCGCTCCAGAGGGATGCA-3′  R 5′-AGTGCAGGGTCCGAGGTATT-3′ | Sangon Biotech Co., Ltd |
| miR-1193 | F 5′-CGGGGATGGTAGACCGGTG-3′  R 5′-AGTGCAGGGTCCGAGGTATT-3′ | Sangon Biotech Co., Ltd |
| miR-7151-5p | F 5′-GCGGATCCATCTCTGCCTGT-3′  R 5′-AGTGCAGGGTCCGAGGTATT-3′ | Sangon Biotech Co., Ltd |
| SESN1 | F 5′-GGACGAGGAACTTGGCATTA-3′  R 5′-ATGCATCTGTGCGTCTTCAC-3′ | Sangon Biotech Co., Ltd |
| CRKL | F 5′-CTGTCGGTGTCCGAGAACTC-3′  R 5′-ATTGGTGGGCTTGGATACCTG-3′ | Sangon Biotech Co., Ltd |
| RAP2C | F 5′-ACCTCAATCATGGCCATACC-3′  R 5′-CAAAGCAGCATCTGTGCAA-3′ | Sangon Biotech Co., Ltd |
| TIMP2 | F 5′-GATGCACATCACCCTCTGTG-3′  R 5′-GTGCCCGTTGATGTTCTTCT-3′ | Sangon Biotech Co., Ltd |
| JUN | F 5′-GCGGACCTTATGGCTACAGT-3′  R 5′-CCCGTTGCTGGACTGGATTA-3′ | Sangon Biotech Co., Ltd |
| CDKN1B | F 5′-ATCACAAACCCCTAGAGGGCA-3′  R 5′-GGGTCTGTAGTAGAACTCGGG-3′ | Sangon Biotech Co., Ltd |

**Table S3** Primer sequences for RT-PCR

| **Primer sequences for RT-PCR** | | |
| --- | --- | --- |
| **Name** | **Sequence** | **Source** |
| RT-miR-429 | 5′-GTCGTATCCAGTGCAGGGTCCGAGGTATTCGCACTGGATA  CGACACGGTT-3′ | Sangon Biotech Co., Ltd |
| RT-miR525-5p/miR-520a-5p | 5′-GTCGTATCCAGTGCAGGGTCCGAGGTATTCGCACTGGATA  CGACAGAAAG-3′ | Sangon Biotech Co., Ltd |
| RT-miR-1193 | 5′-GTCGTATCCAGTGCAGGGTCCGAGGTATTCGCACTGGATA  CGACGCACGT-3′ | Sangon Biotech Co., Ltd |
| RT-miR-7151-5p | 5′-GTCGTATCCAGTGCAGGGTCCGAGGTATTCGCACTGGATA  CGACGCCAAT-3′ | Sangon Biotech Co., Ltd |

**Table S4** ENCORI indicated 11 potential miRNAs bind to circLIFR

| **miRNA** | **MatchGeneID** | **MatchGeneName** | **GeneType** | **TargetSite** | **Alignment** | **Type** | **TDMDScore** | **AgoExpNum** | **CleaveExpNum** | **phyloP** |
| --- | --- | --- | --- | --- | --- | --- | --- | --- | --- | --- |
| **hsa-miR-525-5p** | [NM_001127671](http://www.circbase.org/cgi-bin/singlerecord.cgi?id=NM_001127671) | [LIFR](https://rnasysu.com/encori/agoClipRNA.php?source=circRNA&flag=target&clade=mammal&genome=human&assembly=hg38&miRNA=all&clipNum=1&regionType=None&pval=0.05&clipType=None&deNum=0&target=hsa_circ_0072309#modal) | circRNA | [**chr5:38528766-38528783[-]**](http://genome.ucsc.edu/cgi-bin/hgTracks?db=hg38&lastVirtModeType=default&lastVirtModeExtraState=&virtModeType=default&virtMode=0&nonVirtPosition=&position=chr5%3A38528666-38528883&highlight=hg38.chr5%3A38528766-38528783%23FF5733) | **Target: 5' GGAAA--GCA-CCCUCUGGAA 3'** **\|\|\|\|  \|\|\| \|\|\|\|\|\|\|\|\|** **miRNA : 3' UCUUUCACGUAGGGAGACCUC 5'** | 8mer | 1.5122 | [1](https://rnasysu.com/encori/agoClipRNA.php?source=circRNA&flag=target&clade=mammal&genome=human&assembly=hg38&miRNA=all&clipNum=1&regionType=None&pval=0.05&clipType=None&deNum=0&target=hsa_circ_0072309#modal) | 0 | 1.087 |
| **hsa-miR-520a-5p** | [NM_001127671](http://www.circbase.org/cgi-bin/singlerecord.cgi?id=NM_001127671) | [LIFR](https://rnasysu.com/encori/agoClipRNA.php?source=circRNA&flag=target&clade=mammal&genome=human&assembly=hg38&miRNA=all&clipNum=1&regionType=None&pval=0.05&clipType=None&deNum=0&target=hsa_circ_0072309#modal) | circRNA | [**chr5:38528766-38528783[-]**](http://genome.ucsc.edu/cgi-bin/hgTracks?db=hg38&lastVirtModeType=default&lastVirtModeExtraState=&virtModeType=default&virtMode=0&nonVirtPosition=&position=chr5%3A38528666-38528883&highlight=hg38.chr5%3A38528766-38528783%23FF5733) | **Target: 5' GGAAAGCA---CCCUCUGGAA 3'** **\|\|\|\|\| \|   \|\|\|\|\|\|\|\|\|** **miRNA : 3' UCUUUCAUGAAGGGAGACCUC 5'** | 8mer | 1.4174 | [1](https://rnasysu.com/encori/agoClipRNA.php?source=circRNA&flag=target&clade=mammal&genome=human&assembly=hg38&miRNA=all&clipNum=1&regionType=None&pval=0.05&clipType=None&deNum=0&target=hsa_circ_0072309#modal) | 0 | 1.087 |
| **hsa-miR-7151-5p** | [NM_001127671](http://www.circbase.org/cgi-bin/singlerecord.cgi?id=NM_001127671) | [LIFR](https://rnasysu.com/encori/agoClipRNA.php?source=circRNA&flag=target&clade=mammal&genome=human&assembly=hg38&miRNA=all&clipNum=1&regionType=None&pval=0.05&clipType=None&deNum=0&target=hsa_circ_0072309#modal) | circRNA | [**chr5:38530638-38530659[-]**](http://genome.ucsc.edu/cgi-bin/hgTracks?db=hg38&lastVirtModeType=default&lastVirtModeExtraState=&virtModeType=default&virtMode=0&nonVirtPosition=&position=chr5%3A38530538-38530759&highlight=hg38.chr5%3A38530638-38530659%23FF5733) | **Target: 5' UGCAUUGCA--CAGAUGAUGGAUA 3'** **\|\| \|:\|\|  \|\|\|\| \|\|\|\|\|\|\|** **miRNA : 3' CGGUUAUGUCCGUCU-CUACCUAG 5'** | 8mer | 1.2916 | [1](https://rnasysu.com/encori/agoClipRNA.php?source=circRNA&flag=target&clade=mammal&genome=human&assembly=hg38&miRNA=all&clipNum=1&regionType=None&pval=0.05&clipType=None&deNum=0&target=hsa_circ_0072309#modal) | 0 | 0.098 |
| **hsa-miR-1193** | [NM_001127671](http://www.circbase.org/cgi-bin/singlerecord.cgi?id=NM_001127671) | [LIFR](https://rnasysu.com/encori/agoClipRNA.php?source=circRNA&flag=target&clade=mammal&genome=human&assembly=hg38&miRNA=all&clipNum=1&regionType=None&pval=0.05&clipType=None&deNum=0&target=hsa_circ_0072309#modal) | circRNA | [**chr5:38530611-38530636[-]**](http://genome.ucsc.edu/cgi-bin/hgTracks?db=hg38&lastVirtModeType=default&lastVirtModeExtraState=&virtModeType=default&virtMode=0&nonVirtPosition=&position=chr5%3A38530511-38530736&highlight=hg38.chr5%3A38530611-38530636%23FF5733) | **Target: 5' UUACGUAUGUUUGAAACGACCAUCCU 3'** **\|\|\|\| :::: \|   \| \|\|\|\|\|\|\|** **miRNA : 3' CGUGCA-GUGGCC--AGAUGGUAGGG 5'** | 7mer-m8 | 1.0955 | [1](https://rnasysu.com/encori/agoClipRNA.php?source=circRNA&flag=target&clade=mammal&genome=human&assembly=hg38&miRNA=all&clipNum=1&regionType=None&pval=0.05&clipType=None&deNum=0&target=hsa_circ_0072309#modal) | 0 | 0.110 |
| **hsa-miR-429** | [NM_001127671](http://www.circbase.org/cgi-bin/singlerecord.cgi?id=NM_001127671) | [LIFR](https://rnasysu.com/encori/agoClipRNA.php?source=circRNA&flag=target&clade=mammal&genome=human&assembly=hg38&miRNA=all&clipNum=1&regionType=None&pval=0.05&clipType=None&deNum=0&target=hsa_circ_0072309#modal) | circRNA | [**chr5:38527257-38527279[-]**](http://genome.ucsc.edu/cgi-bin/hgTracks?db=hg38&lastVirtModeType=default&lastVirtModeExtraState=&virtModeType=default&virtMode=0&nonVirtPosition=&position=chr5%3A38527157-38527379&highlight=hg38.chr5%3A38527257-38527279%23FF5733) | **Target: 5' UCAGUUGGAGAAAACCAGUAUUA 3'** **\| \|\|\|  \|  \| \| \|\|\|\|\|\|\|** **miRNA : 3' UGCCAAAAUGGUCU-GUCAUAAU 5'** | 8mer | 0.9756 | [1](https://rnasysu.com/encori/agoClipRNA.php?source=circRNA&flag=target&clade=mammal&genome=human&assembly=hg38&miRNA=all&clipNum=1&regionType=None&pval=0.05&clipType=None&deNum=0&target=hsa_circ_0072309#modal) | 0 | 0.194 |
| hsa-miR-515-5p | [NM_001127671](http://www.circbase.org/cgi-bin/singlerecord.cgi?id=NM_001127671) | [LIFR](https://rnasysu.com/encori/agoClipRNA.php?source=circRNA&flag=target&clade=mammal&genome=human&assembly=hg38&miRNA=all&clipNum=1&regionType=None&pval=0.05&clipType=None&deNum=0&target=hsa_circ_0072309#modal) | circRNA | [**chr5:38527268-38527291[-]**](http://genome.ucsc.edu/cgi-bin/hgTracks?db=hg38&lastVirtModeType=default&lastVirtModeExtraState=&virtModeType=default&virtMode=0&nonVirtPosition=&position=chr5%3A38527168-38527391&highlight=hg38.chr5%3A38527268-38527291%23FF5733) | **Target: 5' CCGUUCUUGUUAUCAGUUGGAGAA 3'** **\| \|    \|\|:\| \|\|  \|\|\|\|\|\|\|** **miRNA : 3' GUCUUUCACGAAAGAAAACCUCUU 5'** | 8mer | 0.8963 | [1](https://rnasysu.com/encori/agoClipRNA.php?source=circRNA&flag=target&clade=mammal&genome=human&assembly=hg38&miRNA=all&clipNum=1&regionType=None&pval=0.05&clipType=None&deNum=0&target=hsa_circ_0072309#modal) | 0 | 0.481 |
| hsa-miR-519e-5p | [NM_001127671](http://www.circbase.org/cgi-bin/singlerecord.cgi?id=NM_001127671) | [LIFR](https://rnasysu.com/encori/agoClipRNA.php?source=circRNA&flag=target&clade=mammal&genome=human&assembly=hg38&miRNA=all&clipNum=1&regionType=None&pval=0.05&clipType=None&deNum=0&target=hsa_circ_0072309#modal) | circRNA | [**chr5:38527268-38527289[-]**](http://genome.ucsc.edu/cgi-bin/hgTracks?db=hg38&lastVirtModeType=default&lastVirtModeExtraState=&virtModeType=default&virtMode=0&nonVirtPosition=&position=chr5%3A38527168-38527389&highlight=hg38.chr5%3A38527268-38527289%23FF5733) | **Target: 5' GUUCUUGUUAUCAGUUGGAGAA 3'** **\|    \|\|:\| :\|  \|\|\|\|\|\|\|** **miRNA : 3' CUUUCACGAGGGAAAACCUCUU 5'** | 8mer | 0.8492 | [1](https://rnasysu.com/encori/agoClipRNA.php?source=circRNA&flag=target&clade=mammal&genome=human&assembly=hg38&miRNA=all&clipNum=1&regionType=None&pval=0.05&clipType=None&deNum=0&target=hsa_circ_0072309#modal) | 0 | 0.543 |
| hsa-miR-624-5p | [NM_001127671](http://www.circbase.org/cgi-bin/singlerecord.cgi?id=NM_001127671) | [LIFR](https://rnasysu.com/encori/agoClipRNA.php?source=circRNA&flag=target&clade=mammal&genome=human&assembly=hg38&miRNA=all&clipNum=1&regionType=None&pval=0.05&clipType=None&deNum=0&target=hsa_circ_0072309#modal) | circRNA | [**chr5:38528751-38528771[-]**](http://genome.ucsc.edu/cgi-bin/hgTracks?db=hg38&lastVirtModeType=default&lastVirtModeExtraState=&virtModeType=default&virtMode=0&nonVirtPosition=&position=chr5%3A38528651-38528871&highlight=hg38.chr5%3A38528751-38528771%23FF5733) | **Target: 5' CUGGAAC-AGGCCGUGGUACUG 3'** **\|\| \|\|\|   \|\|\|\|\|\|\|** **miRNA : 3' ACUUGUGUUCCAUGACCAUGAU 5'** | 7mer-m8 | 0.6699 | [1](https://rnasysu.com/encori/agoClipRNA.php?source=circRNA&flag=target&clade=mammal&genome=human&assembly=hg38&miRNA=all&clipNum=1&regionType=None&pval=0.05&clipType=None&deNum=0&target=hsa_circ_0072309#modal) | 0 | 0.445 |
| hsa-miR-1266-3p | [NM_001127671](http://www.circbase.org/cgi-bin/singlerecord.cgi?id=NM_001127671) | [LIFR](https://rnasysu.com/encori/agoClipRNA.php?source=circRNA&flag=target&clade=mammal&genome=human&assembly=hg38&miRNA=all&clipNum=1&regionType=None&pval=0.05&clipType=None&deNum=0&target=hsa_circ_0072309#modal) | circRNA | [**chr5:38528761-38528782[-]**](http://genome.ucsc.edu/cgi-bin/hgTracks?db=hg38&lastVirtModeType=default&lastVirtModeExtraState=&virtModeType=default&virtMode=0&nonVirtPosition=&position=chr5%3A38528661-38528882&highlight=hg38.chr5%3A38528761-38528782%23FF5733) | **Target: 5' GAAAGCACCCUCUGGAACAGGC 3'** **\|\|     \|:\|\|\|\|\|\|\|** **miRNA : 3' AGGGAGUCCCGUAUCUUGUCCC 5'** | 7mer-m8 | 0.4294 | [1](https://rnasysu.com/encori/agoClipRNA.php?source=circRNA&flag=target&clade=mammal&genome=human&assembly=hg38&miRNA=all&clipNum=1&regionType=None&pval=0.05&clipType=None&deNum=0&target=hsa_circ_0072309#modal) | 0 | 0.753 |
| hsa-miR-200c-3p | [NM_001127671](http://www.circbase.org/cgi-bin/singlerecord.cgi?id=NM_001127671) | [LIFR](https://rnasysu.com/encori/agoClipRNA.php?source=circRNA&flag=target&clade=mammal&genome=human&assembly=hg38&miRNA=all&clipNum=1&regionType=None&pval=0.05&clipType=None&deNum=0&target=hsa_circ_0072309#modal) | circRNA | [**chr5:38527257-38527279[-]**](http://genome.ucsc.edu/cgi-bin/hgTracks?db=hg38&lastVirtModeType=default&lastVirtModeExtraState=&virtModeType=default&virtMode=0&nonVirtPosition=&position=chr5%3A38527157-38527379&highlight=hg38.chr5%3A38527257-38527279%23FF5733) | **Target: 5' UCAGUUGGAGAAAACCAGUAUUA 3'** **\|\|  \|          \|\|\|\|\|\|\|** **miRNA : 3' AGGUAGUAAUGGGCCGUCAUAAU 5'** | 8mer | -0.0255 | [1](https://rnasysu.com/encori/agoClipRNA.php?source=circRNA&flag=target&clade=mammal&genome=human&assembly=hg38&miRNA=all&clipNum=1&regionType=None&pval=0.05&clipType=None&deNum=0&target=hsa_circ_0072309#modal) | 0 | 0.194 |
| hsa-miR-200b-3p | [NM_001127671](http://www.circbase.org/cgi-bin/singlerecord.cgi?id=NM_001127671) | [LIFR](https://rnasysu.com/encori/agoClipRNA.php?source=circRNA&flag=target&clade=mammal&genome=human&assembly=hg38&miRNA=all&clipNum=1&regionType=None&pval=0.05&clipType=None&deNum=0&target=hsa_circ_0072309#modal) | circRNA | [**chr5:38527257-38527278[-]**](http://genome.ucsc.edu/cgi-bin/hgTracks?db=hg38&lastVirtModeType=default&lastVirtModeExtraState=&virtModeType=default&virtMode=0&nonVirtPosition=&position=chr5%3A38527157-38527378&highlight=hg38.chr5%3A38527257-38527278%23FF5733) | **Target: 5' CAGUUGGAGAAAACCAGUAUUA 3'** **\|       \|  \|\|\|\|\|\|\|** **miRNA : 3' AGUAGUAAUGGUCCGUCAUAAU 5'** | 8mer | -0.0284 | [1](https://rnasysu.com/encori/agoClipRNA.php?source=circRNA&flag=target&clade=mammal&genome=human&assembly=hg38&miRNA=all&clipNum=1&regionType=None&pval=0.05&clipType=None&deNum=0&target=hsa_circ_0072309#modal) | 0 | 0.198 |
