# Supplementary material for: Circular RNA circLIFR suppresses papillary thyroid cancer progression by modulating the miR-429/TIMP2 axis
Source: J Cancer Res Clin Oncol. 2024 Jun 25;150(6):323. doi: 10.1007/s00432-024-05839-7 (PMC11196293; doi:10.1007/s00432-024-05839-7)
Supplement: Supplementary file 2 — Supplementary Material 2 [file 432_2024_5839_MOESM2_ESM.docx]

**Supplementary Material**


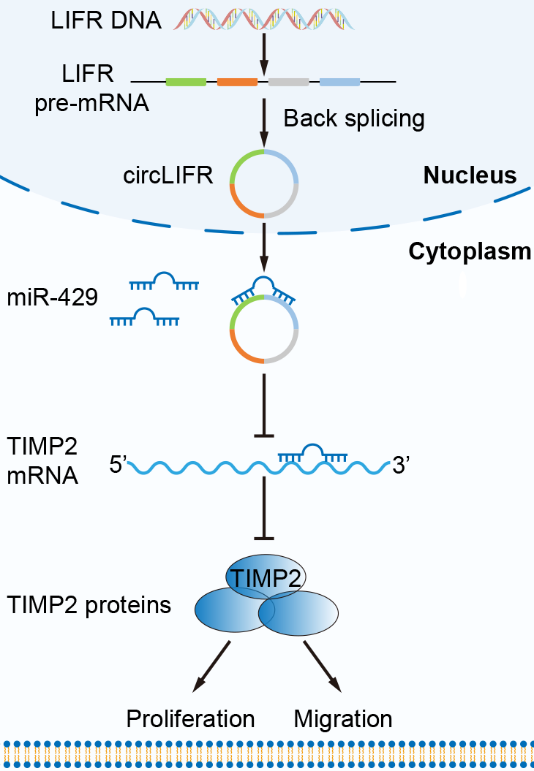


Figure S1 Schematic illustration of circLIFR suppresses papillary thyroid cancer progression by miR-429/TIMP2 axis
